# Optimal impartial correspondences

**DOI:** 10.1007/s00355-025-01631-9

**Published:** 2025-09-18

**Authors:** Javier Cembrano, Felix Fischer, Max Klimm

**Affiliations:** 1 Department of Algorithms and Complexity, Max-Planck-Institute für Informatik, Saarbrücken, Germany; 2 School of Mathematical Sciences, Queen Mary University of London, London, UK; 3 Institute of Mathematics, Technische Universität Berlin, Berlin, Germany

## Abstract

We study mechanisms that select a subset of a set of agents based on nominations among them. The goal is to maximize the minimum number of nominations received by any selected agent, subject to an impartiality constraint that the selection of a particular agent must be independent of the nominations cast by that agent. For situations where each agent casts at most *d* nominations, we give a mechanism that selects at most $d+1$ agents and only selects agents who receive a maximum number of nominations or the maximum number of nominations minus one. We then show that this is best possible in the sense that no impartial mechanism can only select agents receiving a maximum number of nominations, even without any restrictions on the number of selected agents. We finally establish the following trade-off between the maximum number of agents selected and the minimum number of nominations for any selected agent when there are no constraints on the number of nominations each agent can cast: when selecting at most *k* agents out of *n*, it is possible to only select agents that receive at least the maximum number of nominations minus $\big \lfloor \frac{n-2}{k-1} \big \rfloor +1$.

## Introduction

The goal in impartial selection is to select agents from a set based on nominations among the agents. The axiom of *impartiality* requires the selection of a particular agent to be independent of the nominations cast by that agent. This axiom is a natural requirement in situations where agents nominate one another for selection and are willing to offer their true opinions on other agents as long as doing so does not affect their own chances of being selected.

The impartial selection of a single agent is subject to strong impossibility results. In a setting where each agent nominates exactly one other agent, every impartial selection rule violates one of two basic axioms: *positive unanimity*, that an agent nominated by all other agents must be selected, and *negative unanimity*, that an agent not receiving any nominations must not be selected (Holzman and Moulin 2013). When agents can cast nominations for an arbitrary number of other agents, all impartial selection rules violate an even weaker axiom, that an agent not receiving any nominations must not be selected when there exists an agent nominated by all other agents (Cembrano et al. 2024). Similar impossibilities remain in place when any fixed number of agents is to be selected. When each agent nominates at most one other agent, every impartial rule selecting a fixed number $k\in \{1,\dots ,n-1\}$ out of a total of *n* agents sometimes fails to select any nominated agent when such an agent exists (Alon et al. 2011).

The impossibility results can be overcome by allowing the selection of a variable number of agents.^1^ For a setting where each agent nominates exactly one other agent, Tamura and Ohseto (2014) proposed an impartial mechanism, *plurality with runners-up*, that satisfies appropriate versions of positive and negative unanimity and may select any number $k\in \{1,\dots ,n\}$ out of a total of *n* agents. From a practical point of view, the need to select a variable number of agents should not necessarily be a cause for concern. Indeed, situations in the real world requiring impartial selection often allow for a certain degree of flexibility in the number of selected agents. The exact number of papers accepted to an academic conference is usually not fixed in advance but can be varied depending on the number and quality of submissions. Best paper awards at conferences are often given in overlapping categories, and some awards may only be given if this is warranted by the field of candidates. The Fields medal is awarded every four years to two, three, or four mathematicians under the age of 40. Examples at the more extreme end of the spectrum of flexibility include the award of job titles such as vice president or deputy vice-principal. Such titles can often be given to a large number of individuals at a negligible cost per individual.

In all of these examples, selection is associated with quality as well as distinction. It thus makes sense to require that only agents with a certain number of nominations are selected relative to the maximum number of nominations for any agent, and that not too many agents are selected simultaneously. Call a selection mechanism $\alpha$-*optimal* for $\alpha \in \mathbb {N}$ if the difference between the maximum number of nominations for any agent and the minimum number of nominations for any selected agent is always at most $\alpha$. Call a mechanism *decisive* if it never selects all agents, and *k*-decisive for $k\in \mathbb {N}$ if it always selects at most *k* agents. In terms of quality one might hope for 0-optimality, meaning that only agents receiving a maximum number of nominations can be selected. It turns out, however, and is stated formally as Proposition 1, that 0-optimality and impartiality are incompatible. When searching for a rule that is impartial and $\alpha$-optimal for a small positive value of $\alpha$, it is interesting to revisit plurality with runners-up (Tamura and Ohseto 2014). The rule selects any agent who receives a maximum number of nominations; if there is a unique such agent, any agent who receives one fewer nomination and who nominates the unique agent with a maximum number of nominations is selected as well. The rule is clearly 1-optimal, and it is not difficult to show that it is also impartial. It is not decisive, but under the assumption that each agent casts only a single nomination it can be made 2-decisive by breaking ties according to a fixed ordering of the agents (Tamura and Ohseto 2014). As the combination of 1-decisiveness and 1-optimality is ruled out by known impossibilities, this provides the best possible tradeoff between optimality and decisiveness under the assumption. When agents can cast multiple nominations, the rule loses its unique combination of properties and is not easily repaired. Voting systems in which voters are allowed to nominate more than one candidate—known as *approval voting*—have received considerable attention in economics and computer science; the books by Brams and Fishburn (2007) and Laslier and Sanver (2010) provide an overview of this area. In our setting, where voters and candidates coincide, approval voting has clear practical relevance: for example, referees may write reports for multiple papers, and employees may endorse multiple colleagues for promotion.

### Our contribution

We investigate impartial, $\alpha$-optimal, and *k*-decisive mechanisms in settings where agents may cast multiple nominations. Our first result provides a generalization of the result of Tamura and Ohseto to this setting: for the case where each agent casts at most *d* nominations, we give an impartial mechanism that is 1-optimal and $(d+1)$-decisive, i.e., it selects at most $d+1$ agents and only selects agents receiving a maximal number of nominations or a maximal number of nominations minus 1. For the particular case of an unrestricted number of nominations, namely $d = n-1$, this may lead to all agents being selected so that the resulting mechanism is not decisive. However, we can show that also in that case, there is an impartial, 1-optimal, and decisive mechanism.

Our second result establishes that 1-optimality is best possible, thus ruling out the existence of impartial mechanisms that only select agents with maximum number of nominations. In fact, we provide in Theorem 3 a stronger result showing that in a setting where every agent casts at most *d* nominations, any impartial and 1-optimal mechanism has to sometimes not select any of the agents receiving a maximum number of nominations, or has to sometimes select only one of the agents receiving a maximum number of nominations and at least $\big \lfloor \frac{d+1}{2}\big \rfloor$ agents with fewer nominations.

Our third result provides a trade-off between the maximum number of agents selected and the minimum number of nominations received by any selected agent when each agent can cast an arbitrary number of nominations: for *k*-decisive mechanisms selecting among a set of *n* agents, we can guarantee $\alpha$-optimality for $\alpha =\big \lfloor \frac{n-2}{k-1}\big \rfloor +1$. This is achieved by removing a suitable subset of the nominations before plurality with runners-up is applied, in order to guarantee impartiality while selecting fewer agents. We do not know whether this last result is tight and leave open the interesting question for the optimal trade-off between the number and quality of selected agents.

### Related work

Impartiality as a property of an economic mechanism was introduced by Clippel et al. (2008). Alon et al. (2011) and Holzman and Moulin (2013) introduced the impartial selection problem and formalized it by a nomination graph where vertices correspond to agents and there is a directed edge from one agent to another if the first agent nominates the latter. The outdegree of a vertex in this graph then corresponds to the number of nominations that the agent casts and the indegree of a vertex corresponds to the number of nominations that the agent receives. Whereas Holzman and Moulin gave axiomatic characterizations for mechanisms selecting a single vertex when all outdegrees are equal to one, Alon et al. studied the ability of impartial mechanisms to approximate the maximum indegree for any fixed number of vertices when there are no limitations on outdegrees.

Both sets of authors obtained strong impossibility results, which a significant amount of follow-up work has since sought to overcome. Randomized mechanisms providing non-trivial multiplicative guarantees had already been proposed by Alon et al. (2011) and Fischer and Klimm (2015) subsequently achieved the best possible such guarantee for the selection of one vertex. Randomized mechanisms have been also studied from an axiomatic point of view by Mackenzie (2015, 2020). Starting from the observation that worst-case instances for randomized mechanisms have small indegrees, Bousquet et al. (2014) developed a mechanism that is asymptotically optimal as the maximum indegree grows, and Caragiannis et al. (2022, 2023) initiated the study of mechanisms providing additive rather than multiplicative guarantees. More specifically, Caragiannis et al. (2022) devised a randomized impartial mechanism selecting at most one vertex among *n* vertices and that is in expectation $\alpha$-optimal where $\alpha \in \Theta (n^{2/3} \ln ^{1/3} n)$. For the special case that the outdegree of each vertex is one, they provided a randomized impartial mechanism selecting a single vertex and that is $\alpha$-optimal with $\alpha \in \Theta (\sqrt{n})$. The latter optimality guarantee was obtained by Cembrano et al. (2024) for a deterministic mechanism in cases where the maximum outdegree is bounded by a constant; they further established that non-trivial guarantees can be obtained with unbounded outdegrees. The results of this paper show that much smaller bounds on the optimality can be achieved by mechanisms that are allowed to select more than one vertex.

Bjelde et al. (2017) gave randomized mechanisms with improved multiplicative guarantees for the selection of more than one vertex and observed that when selecting at most *k* vertices rather than exactly *k*, deterministic mechanisms can in fact achieve non-trivial guarantees. Cembrano et al. (2023) gave deterministic mechanisms with improved approximation guarantees. An axiomatic study of Tamura and Ohseto (2014) for the outdegree-one case came to the same conclusion: when allowing for the selection of a varying number of vertices, the impossibility result of Holzman and Moulin no longer holds. Tamura (2016) subsequently characterized the mechanism proposed by Tamura and Ohseto, which in some cases selects all vertices, as the unique minimal mechanism satisfying impartiality, anonymity, symmetry, and monotonicity.

Impartial mechanisms have finally been proposed for various problems other than selection, including peer review (Aziz et al. 2019; Kurokawa et al. 2015; Mattei et al. 2020; Xu et al. 2019), rank aggregation (Kahng et al. 2018), progeny maximization (Babichenko et al. 2020; Zhang et al. 2021), and network centralities (Wąs et al. 2019).

## Preliminaries

For $n\in \mathbb {N}$, let $[n]=\{1,2,\dots ,n\}$, and let

$$\begin{aligned} \mathcal {G}_n = \Bigg \{(V,E):V=[n],E \subseteq (V\times V) {\setminus } \bigcup _{v\in V}\{(v,v)\}\Bigg \} \end{aligned}$$

be the set of directed graphs with *n* vertices and no loops. Let $\mathcal {G}= \bigcup _{n\in \mathbb {N}} \mathcal {G}_n$. For $G=(V,E)\in \mathcal {G}$ and $v\in V$, let $N^+(v, G)=\{u\in V: (v,u) \in E\}$ be the out-neighborhood and $N^-(v, G)=\{u\in V:(u,v)\in E\}$ the in-neighborhood of *v* in *G*. Let $\delta ^+(v,G)=|N^+(v,G)|$ and $\delta ^-(v,G)=|N^-(v,G)|$ be the outdegree and indegree of *v* in *G*, and $\Delta (G) = \max \{\delta ^-(v, G): v\in V\}$ the maximum indegree of any vertex in *G*. When the graph is clear from the context, we will sometimes drop *G* from the notation and write $N^+(v)$, $N^-(v)$, $\delta ^+(v)$, $\delta ^-(v)$, and $\Delta$. Let ${{\,\textrm{top}\,}}(G)=\max \{v\in V:\delta ^-(v,G)=\Delta (G)\}$ be the vertex of *G* with the largest index among those with maximum indegree. For $n\in \mathbb {N}$ and $d\in [n-1]$, let

$$\begin{aligned} \mathcal {G}_n(d) = \{(V, E)\in \mathcal {G}_n: \delta ^+(v,G)\le d{\text { for all }}v\in V\} \end{aligned}$$

be the set of graphs in $\mathcal {G}_n$ with outdegrees at most *d*, and $\mathcal {G}(d) = \bigcup _{n\in \mathbb {N}} \mathcal {G}_n(d)$.

A *selection mechanism* is then given by a family of functions $f:\mathcal {G}_n \rightarrow 2^{[n]}{\setminus } \{\emptyset \}$, one for each $n\in \mathbb {N}$, mapping each graph to a non-empty subset of its vertices.^2^ In a slight abuse of notation, we will use *f* to refer to both the mechanism and to individual functions of the family. Given $G=(V,E)\in \mathcal {G}$ and $v\in V$, let

$$\mathcal {N}_v(G)=\{(V,E')\in \mathcal {G}:\ E{\setminus }(\{v\}\times V)=E'{\setminus }(\{v\}\times V)\}$$

be the set neighboring graphs of *G* with respect to *v*, in the sense that they can be obtained from *G* by changing the outgoing edges of *v*. The central axiom considered in this paper is that of impartiality. It requires the selection of a vertex to be independent of its outgoing edges.

### Definition 1

(*Impartiality*) A mechanism *f* is *impartial* on $\mathcal {G}'\subseteq \mathcal {G}$ if on this set of graphs the outgoing edges of a vertex have no influence on its selection, i.e., if for every graph $G = (V,E)\in \mathcal {G}'$, $v\in V$, and $G'\in \mathcal {G}'\cap \mathcal {N}_v(G)$, it holds that $f(G)\cap \{v\}=f(G')\cap \{v\}$.

We measure the quality of a selection as the gap between the maximum indegree of any vertex and the minimum indegree of any selected vertex.

### Definition 2

($\alpha$-*optimality*) A mechanism *f* is $\alpha$-*optimal* on $\mathcal {G}'\subseteq \mathcal {G}$, for $\alpha \in \mathbb {N}$, if on this set of graphs the mechanism exclusively selects vertices with indegree equal to the maximum indegree minus $\alpha$ or higher, i.e., if for every graph $G \in \mathcal {G}'$, $v\in f(G)$ implies $\delta ^-(v,G)\ge \Delta (G)-\alpha$.

A 0-optimal mechanism will be referred to simply as *optimal*. To make sure that the selection process is informative, we are finally interested in imposing an upper bound on the number of selected vertices.

### Definition 3

(*k*-*decisiveness*) A mechanism *f* is *k*-*decisive* on $\mathcal {G}'\subseteq \mathcal {G}$, for $k\in [n]$, if on this set of graphs the mechanism selects at most *k* vertices, i.e., if for every graph $G \in \mathcal {G}'$, it holds that $|f(G)|\le k$.

If *f* is $(n-1)$-decisive on $\mathcal {G}'$, i.e., it never selects all vertices, we simply call it *decisive* on $\mathcal {G}'$.

## Impartiality and near-optimality

Impartiality and optimality are two very natural requirements for selection mechanisms, but they are incompatible independently of the maximum outdegree. This is stated formally in the following simple proposition.

### Proposition 1

For every $n\in \mathbb {N}$ and $d\in [n-1]$, there is no selection mechanism that is both impartial and optimal on $\mathcal {G}_n(d)$.

### Proof

**Fig. 1:**
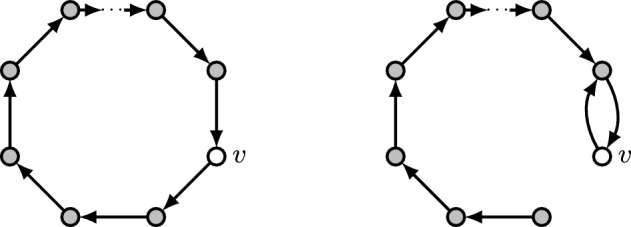
Nomination graphs considered in the proof of Proposition 1. The vertex drawn in white has to be selected

Let $n\in \mathbb {N}$ and $d\in [n-1]$ be arbitrary values. Suppose *f* is an impartial and optimal selection mechanism on $\mathcal {G}_n(d)$. Consider $V=[n]$ and the graph $G=(V,E)$ consisting of an *n*-cycle, i.e., $E=\{(u,u+1): u\in [n-1]\}\cup \{(n,1)\}$; see the left graph in Fig. 1. Fixing an arbitrary vertex $v\in f(G)$, we now define the graph $G'=(V,E')$ constructed by deleting *v*’s outgoing edge and adding an edge from *v* to its in-neighbor in *G*. Formally, defining $u=v-1$ if $v\not =1$ and $u=n$ if $v=1$, we define $E'=E{\setminus } (\{v\} \times V) \cup \{(v,u)\}$; see also the right graph in Fig. 1. It is clear that $G'\in \mathcal {G}_n(d)\cap \mathcal {N}_v(G)$, thus impartiality implies $v\in f(G')$. However, since $\delta ^-(v,G')=1<2=\delta ^-(u,G')$, optimality of *f* implies $v\not \in f(G')$, a contradiction. $\square$

Since it is not possible to achieve impartiality and optimality, it is natural to relax the demanding optimality condition to allow selecting vertices with indegree $\Delta -1$, as captured by our definition of 1-optimality. These two axioms have been shown to be compatible for the case of graphs with outdegree one by Tamura and Ohseto (2014), who proposed a mechanism they called *plurality with runners-up*. The mechanism, which we describe formally in Algorithm 1, selects all vertices with maximum indegree; if there is a unique such vertex, then any vertex with an outgoing edge to that vertex whose indegree is smaller by one is selected as well. The idea behind this mechanism is that vertices in the latter category would be among those with maximum indegree if their outgoing edge was deleted, and thus any impartial mechanism seeking to select the vertices with maximum indegree would also have to select those vertices. Plurality with runners-up is impartial on $\mathcal {G}(1)$, and in any graph with *n* vertices selects between 1 and *n* vertices whose indegree is equal to the maximum indegree or the maximum indegree minus one. It is thus an impartial and 1-optimal selection mechanism on $\mathcal {G}(1)$. It is natural to ask whether a similar result can be obtained for more general settings.

**Algorithm 1:**
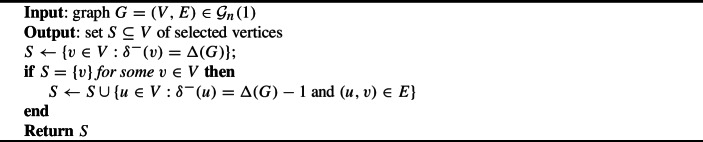
Plurality with runners-up

While Tamura and Ohseto do not limit the maximum number of selected vertices, they discuss briefly a modification of their mechanism that retains impartiality and 1-optimality but selects at most 2 vertices. Instead of selecting all vertices with maximum indegree, the modified mechanism breaks ties in favor of a single maximum-indegree vertex using a fixed ordering of the vertices. In order to guarantee impartiality, the modified mechanism then also selects any vertex that would be selected in the graph obtained by deleting the outgoing edge of that vertex. Since every vertex has at most one outgoing edge, at most one vertex can decrease the indegree of the originally selected vertex and become the maximum-indegree vertex with the highest priority, which implies that at most one additional vertex is selected. There thus exists an impartial, 1-optimal, and *k*-decisive selection mechanism on $\mathcal {G}(1)$ for every $k\ge 2$. Our first result generalizes this modified mechanism to settings with arbitrary outdegrees, as long as the maximum number of selected vertices is large enough. To this end we show that when the maximum outdegree is *d*, to achieve impartiality, at most *d* vertices have to be selected in addition to the one with maximum indegree and highest priority.^3^ We formally describe the resulting mechanism in Algorithm 2, and will refer to it as *asymmetric plurality with runners-up* and denote its output on graph *G* by $f_{\text {pr}}(G)$. We obtain the following theorem, which generalizes the known result for the outdegree-one case.

**Algorithm 2:**
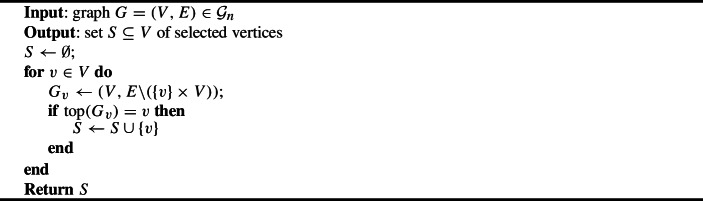
Asymmetric plurality with runners-up ($f_{\text {pr}}$)

### Theorem 1

For every $n\in \mathbb {N},\ d\in [n-1]$, and $k\in \{d+1,\ldots ,n\}$, there exists a selection mechanism that is impartial, 1-optimal, and *k*-decisive on $\mathcal {G}_n(d)$.

To prove the theorem, we will be interested in the following in comparing vertices both according to their indegree and to their index. We will use generalized inequality symbols $\prec$ and $\succ$, their non-strict variants $\preceq$ and $\succeq$, and the operators $\max$ and $\min$, to denote the lexicographic order among pairs of the form $(\delta ^-(v),v)$. The following lemma characterizes the structure of the set of vertices selected by asymmetric plurality with runners-up, and provides the main technical ingredient for the proof of Theorem 1.

### Lemma 1

Let $G=(V,E)\in \mathcal {G}$ and $v\in V$. Then, $v\in f_{\text {pr}}(G)$ if and only if

- for every $w\in V$ with $(\delta ^-(w),w)\succ (\delta ^-(v),v)$ it holds that $(v,w)\in E$; and
- one of the following holds:
  (i) $$\delta ^-(v)=\Delta (G)$$; or
  (ii) $\delta ^-(v)=\Delta (G)-1$ and $v>w$ for every $w\in V$ with $\delta ^-(w)=\Delta (G)$.

### Proof

We first show that, if we have $v\in f_{\text {pr}}(G)$ for a given graph *G*, then (a) and (b) follow. Let $G=(V,E)\in \mathcal {G}$ and $v\in f_{\text {pr}}(G)$. To see (a), suppose there is a vertex $w\in V$ with $(\delta ^-(w,G),w)\succ (\delta ^-(v,G),v)$. Denoting $G_v = (V,E{\setminus } (\{v\}\times V))$, we have $v={{\,\textrm{top}\,}}(G_v)$ due to $v\in f_{\text {pr}}(G)$. This implies $(\delta ^-(v,G_v),v)\succ (\delta ^-(w,G_v),w)$ and therefore $\delta ^-(w,G)>\delta ^-(w,G_v)$, because $\delta ^-(v,G)=\delta ^-(v,G_v)$. Since *G* and $G_v$ only differ in the outgoing edges of *v*, we conclude that $(v,w)\in E$. To prove (b), we note that for every $w\in V$ we have

1 $$\begin{aligned} (\delta ^-(v,G),v)=(\delta ^-(v,G_v),v)\succ (\delta ^-(w,G_v),w)\succeq (\delta ^-(w,G)-1,w), \end{aligned}$$

where the last inequality comes from the fact that each vertex has at most one incoming edge from *v*. If there is no $w\in V{\setminus } \{v\}$ with $\delta ^-(w)=\Delta (G)$, the maximum indegree must be that of *v*, so $\delta ^-(v)=\Delta (G)$ and (i) follows. Otherwise, for each $w\in V{\setminus } \{v\}$ with $\delta ^-(w)=\Delta (G)$, (1) yields $(\delta ^-(v,G),v)\succ (\Delta (G)-1,w)$. We conclude that either $\delta ^-(v,G)>\Delta (G)-1$, which implies (i), or both $\delta ^-(v)=\Delta (G)-1$ and $v>w$, which implies (ii).

We now prove the other direction. Let $G=(V,E)\in \mathcal {G}$ and $v\in V$ be such that both (a) and (b) hold. Let $G_v=(V,E{\setminus } (\{v\}\times V))$. We have to show that ${{\,\textrm{top}\,}}(G_v)=v$, i.e., that for every $w\in V{\setminus } \{v\},\ (\delta ^-(v, G_v), v)\succ (\delta ^-(w,G_v),w)$. Let *w* be a vertex in $V{\setminus } \{v\}$. If $(\delta ^-(v, G), v)\succ (\delta ^-(w,G),w)$, we can conclude immediately since $\delta ^-(v, G_v)=\delta ^-(v, G)$ and $\delta ^-(w,G_v)\le \delta ^-(w,G)$. Otherwise, we know from (a) that $(v,w)\in E$ and, therefore, $\delta ^-(w,G_v)= \delta ^-(w,G)-1$. If *v* satisfies (i), this yields

$$\begin{aligned} \delta ^-(v, G_v) = \delta ^-(v, G) = \Delta (G) \ge \delta ^-(w,G) = \delta ^-(w,G_v)+1, \end{aligned}$$

so $(\delta ^-(v, G_v), v)\succ (\delta ^-(w,G_v),w)$. On the other hand, if *v* satisfies (ii), then

$$\begin{aligned} \delta ^-(v, G_v) = \delta ^-(v, G) = \Delta (G)-1 \ge \delta ^-(w,G)-1 = \delta ^-(w,G_v), \end{aligned}$$

and $v>w$ implies $(\delta ^-(v, G_v), v)\succ (\delta ^-(w,G_v),w)$ as well. $\square$

**Fig. 2:**
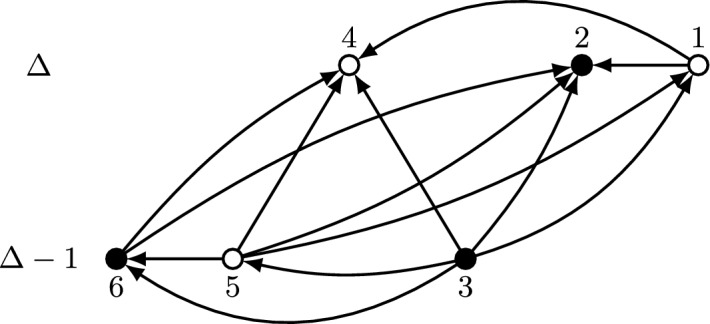
Example of a set of vertices selected by asymmetric plurality with runners-up. Vertices are arranged vertically according to indegree and horizontally according to index, so that vertices on the left are favored in case of ties. The vertices selected by the mechanism are drawn in white and those not selected in black. Vertices with indegree below $\Delta -1$, as well as edges incident to such vertices, are not shown. Denoting the graph as $G=(V,E)$, and letting $G_v = (V,E{\setminus } (\{v\}\times V))$ for each vertex *v*, the selected vertices *v* are those for which ${{\,\textrm{top}\,}}(G_v)=v$. Specifically, vertices 2, 3, and 6 are not selected because ${{\,\textrm{top}\,}}(G_2)=4$, ${{\,\textrm{top}\,}}(G_3)=4$, and ${{\,\textrm{top}\,}}(G_6)=1$

Observe that Lemma 1 implies in particular that ${{\,\textrm{top}\,}}(G)\in f_{\text {pr}}(G)$ for every graph *G*. Figure 2 provides an example of the characterization given by Lemma 1, in terms of indegrees, tie-breaking order, and edges among selected vertices.

We are now ready to prove Theorem 1.

### Proof of Theorem 1

We show that for every $n\in \mathbb {N}$ and $d\in [n-1]$, asymmetric plurality with runners-up is an impartial and 1-optimal selection mechanism on $\mathcal {G}_n(d)$, and that for every $G=(V,E)\in \mathcal {G}_n(d)$, it selects at least one and at most $d+1$ vertices. If this is the case, then for every $k\in \{d+1,\ldots ,n\}$ asymmetric plurality with runners-up would satisfy the statement of the theorem. Therefore, let *n* and *d* be as mentioned.

The fact that asymmetric plurality with runners-up is a selection mechanism is clear from the fact that for every $G\in \mathcal {G}_n(d),~ f_{\text {pr}}(G)\ne \emptyset$ since Lemma 1 implies that ${{\,\textrm{top}\,}}(G)\in f_{\text {pr}}(G)$.

Impartiality follows from the definition of the mechanism, because the outgoing edges of a vertex are not taken into account when deciding whether the vertex is in the selected set or not. If we let $G=(V,E),\ v\in V$, and $G'=(V,E')\in \mathcal {N}_v(G)$, then the graphs $G_v$ and $G'_v$ constructed when running the mechanism with each of these graphs *G* and $G'$ as an input, respectively, are the same because by definition of $\mathcal {N}_v(G)$ we have $E{\setminus }(\{v\}\times V)=E'{\setminus }(\{v\}\times V)$. Since $v\in f_{\text {pr}}(G)\Leftrightarrow {{\,\textrm{top}\,}}(G_v)=v$, and $v\in f_{\text {pr}}(G')\Leftrightarrow {{\,\textrm{top}\,}}(G'_v)=v$, we conclude that $v\in f_{\text {pr}}(G) \Leftrightarrow v\in f_{\text {pr}}(G')$.

The 1-optimality follows directly from Lemma 1 since this lemma implies that for every $G\in \mathcal {G}_n(d)$ and every $v\in f_{\text {pr}}(G),\ \delta ^-(v)\ge \Delta (G)-1$.

Finally, let $G=(V,E)\in \mathcal {G}_n(d)$, and suppose that $|f_{\text {pr}}(G)|>d+1$. If we denote $v_L=\arg \min \{(\delta ^-(v),v): v\in f_{\text {pr}}(G)\}$, from Lemma 1 we know that $(v_L,w)\in E$ for every $w\in V$ with $(\delta ^-(w),w)\succ (\delta ^-(v_L),v_L)$, thus $\delta ^+(v_L)\ge |f_{\text {pr}}(G)|-1 > d$, a contradiction. We conclude that $|f_{\text {pr}}(G)|\le d+1$. $\square$

The following result, concerning mechanisms that may select an arbitrary number of vertices, follows immediately from Theorem 1.

### Corollary 1

There exists an impartial and 1-optimal selection mechanism on $\mathcal {G}$.

On $\mathcal {G}_n$, i.e., in the case of unbounded outdegrees, the mechanism selects all vertices for some input graphs: it is not decisive. We can, however, avoid this drawback through a more intricate version of asymmetric plurality with runners-up, which we call *asymmetric plurality with runners-up and pivotal vertices*. We formally describe this mechanism in Algorithm 3 and denote its output for graph *G* by $f_{\text {prp}}(G)$. Given a graph $G=(V,E)$, call a vertex $u\in f_{\text {pr}}(G)$ *pivotal* for $v\in f_{\text {pr}}(G)$ if there exists a graph $G_{uv}\in \mathcal {N}_u(G)$ such that $v\notin f_{\text {pr}}(G_{uv})$, i.e., if the outgoing edges of *u* can be changed in such a way that *v* is no longer selected by asymmetric plurality with runners-up. Asymmetric plurality with runners-up and pivotal vertices then selects every vertex in $f_{\text {pr}}(G)$ that is pivotal for every other vertex in $f_{\text {pr}}(G)$. The mechanism turns out to inherit impartiality and 1-optimality, and to never select all vertices.

**Algorithm 3:**
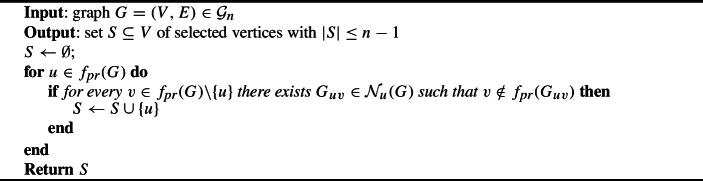
Asymmetric plurality with runners-up and pivotal vertices ($f_{\text {prp}}$)

### Theorem 2

There exists a selection mechanism that is impartial, 1-optimal, and decisive on $\mathcal {G}$.

While impartiality and 1-optimality of asymmetric plurality with runners-up and pivotal vertices are easy to see, the fact that it selects at least 1 and at most $n-1$ vertices on any graph $G\in \mathcal {G}_n$ turns out to be more difficult to show. To prove the former, we distinguish two cases depending on whether ${{\,\textrm{top}\,}}(G)$ has outgoing edges to all other vertices in $f_{\text {pr}}(G)$ or not. If this is the case, then ${{\,\textrm{top}\,}}(G)$ can remove all other vertices from $f_{\text {pr}}(G)$ by deleting its outgoing edges and is selected by the mechanism; otherwise, we prove that the vertex $v\in f_{\text {pr}}(G)$ that minimizes $(\delta ^-(v,G),v)$ is selected. Decisiveness is shown separately for the complete graph—where we show that only the vertex with the largest index is selected—and for any other graph with all indegrees in $\{n-2,n-1\}$—where we show that vertices with outdegree $n-2$ or smaller are not selected.

### Proof of Theorem 2

We show that for every $n\in \mathbb {N}$, asymmetric plurality with runners-up and pivotal vertices is an impartial and 1-optimal selection mechanism on $\mathcal {G}_n$ and that, for every $G=(V,E)\in \mathcal {G}_n$, it selects at most $n-1$ vertices. Let $n\in \mathbb {N}$ be an arbitrary value.

We first show that asymmetric plurality with runners-up and pivotal vertices is indeed a selection mechanism, i.e., that it always selects at least one vertex. To this purpose, we let $G=(V,E)\in \mathcal {G}_n$ be a graph and introduce some additional notation. Let $S^i=\{v\in f_{\text {pr}}(G):\delta ^-(v)=\Delta (G)-i\}$ and $n^i=|S^i|$ for $i\in \{0,1\}$, and denote

$$\begin{aligned} v_H&=\arg \max \{(\delta ^-(v,G), v): v\in f_{\text {pr}}(G)\}={{\,\textrm{top}\,}}(G),\\ v_L&=\arg \min \{(\delta ^-(v,G), v): v\in f_{\text {pr}}(G)\}. \end{aligned}$$

From Lemma 1, we know that $f_{\text {pr}}(G)= S^0\cup S^1$, that $(v_L,v)\in E$ for every $v\in f_{\text {pr}}(G){\setminus }\{v_L\}$, and that $u>v$ for each $u\in S^1,\ v\in S^0$. We now distinguish two cases according to the edges between vertices in $f_{\text {pr}}(G)$.

**Fig. 3:**
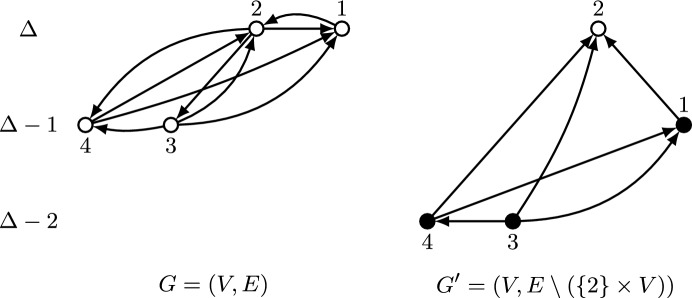
Illustration of the fact that the set of vertices selected by asymmetric plurality with runners-up and pivotal vertices is non-empty if $(v_H,v)\in E$ for every $v\in f_{\text {pr}}(G){\setminus } \{v_H\}$. Vertices are arranged vertically according to indegree and horizontally according to index, so that vertices on the left are favored in case of ties. Vertices with indegree below $\Delta -1$, as well as edges incident to such vertices, are not shown. Vertices selected by asymmetric plurality with runners-up are drawn in white; vertices not selected by that mechanism are drawn in black. Denoting the graph on the left as $G=(V,E)$, where $v_H=2$, and defining $G'=(V,E{\setminus } (\{2\}\times V))\in \mathcal {N}_2(G)$, we have that $\{1,3,4\}\cap f_{\text {pr}}(G')=\emptyset$, and thus $2\in f_{\text {prp}}(G)$

If $(v_H,v)\in E$ for every $v\in f_{\text {pr}}(G){\setminus } \{v_H\}$, we define $G'=(V,E{\setminus } (\{v_H\}\times V))\in \mathcal {N}_{v_H}(G)$ and we claim that $v\notin f_{\text {pr}}(G')$ for every $v\in f_{\text {pr}}(G){\setminus } \{v_H\}$. If this is true, it is clear that $v_H\in f_{\text {prp}}(G)$ and thus $f_{\text {prp}}(G)\ne \emptyset$. We now prove the claim. First, note that $v_H\in f_{\text {pr}}(G')$ since $v_H={{\,\textrm{top}\,}}(G')$ and Lemma 1 ensures ${{\,\textrm{top}\,}}(G')\in f_{\text {pr}}(G')$. This comes from the fact that $\delta ^-(v_H,G')=\delta ^-(v_H,G)$ and $\delta ^-(v,G')\le \delta ^-(v,G)$ for every $v\in V{\setminus } \{v_H\}$, along with $v_H={{\,\textrm{top}\,}}(G)$. Moreover, for every $v\in S^0{\setminus } \{v_H\}$ it holds that

$$\begin{aligned} \delta ^-(v,G')=\delta ^-(v,G)-1 = \delta ^-(v_H,G')-1= \Delta (G')-1 \end{aligned}$$

and $v<v_H$, so condition (b) in Lemma 1 does not hold for *v* and thus $v\notin f_{\text {pr}}(G')$. Analogously, for every $v\in S^1$ it holds that

$$\begin{aligned} \delta ^-(v,G')=\delta ^-(v,G)-1 = \delta ^-(v_H,G')-2= \Delta (G')-2, \end{aligned}$$

so condition (b) in Lemma 1 does not hold for *v* either, and thus $v\notin f_{\text {pr}}(G')$. This allows to conclude the claim and the fact that $f_{\text {prp}}(G)$ is non-empty for this case. This argument is illustrated in Fig. 3.

**Fig. 4:**
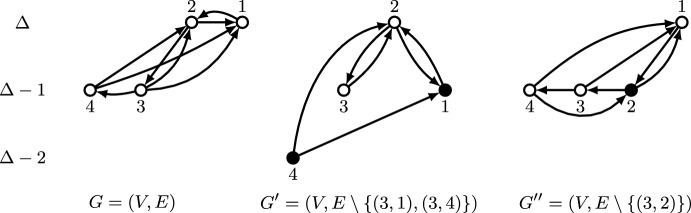
Illustration of the fact that the set of vertices selected by asymmetric plurality with runners-up and pivotal vertices is non-empty if $(v_H,\bar{v})\notin E$ for some $\bar{v}\in f_{\text {pr}}(G){\setminus } \{v_H\}$. Vertices are arranged vertically according to indegree and horizontally according to index, so that vertices on the left are favored in case of ties. Vertices with indegree below $\Delta -1$, as well as edges incident to such vertices, are not shown. Vertices selected by asymmetric plurality with runners-up are drawn in white; vertices not selected are drawn in black. For $G=(V,E)$ we have $v_H=2$, $v_L=3$, and $\bar{v}=4$. Thus, $\{1,4\}\cap f_{\text {pr}}(G')=\emptyset$ for $G'=(V,E{\setminus } \{(3,1), (3,4)\})\in \mathcal {N}_3(G)$ and $2\notin f_{\text {pr}}(G'')$ for $G''=(V,E{\setminus } \{(3,2)\})\in \mathcal {N}_3(G)$. We conclude that $3\in f_{\text {prp}}(G)$

Now we consider the case where there is a vertex $\bar{v}\in f_{\text {pr}}(G){\setminus } \{v_H\}$ such that $(v_H, \bar{v})\notin E$, and we claim that defining $G'=(V,(E{\setminus } (\{v_L\}\times V))\cup (v_L,v_H))\in \mathcal {N}_{v_L}(G)$ it holds $v\notin f_{\text {pr}}(G')$ for every $v\in f_{\text {pr}}(G){\setminus } \{v_L, v_H\}$, whereas defining $G''=(V,E{\setminus } (v_L,v_H))\in \mathcal {N}_{v_L}(G)$ it holds $v_H\notin f_{\text {pr}}(G'')$. If this is true, then $v_L\in f_{\text {prp}}(G)$ and $f_{\text {prp}}(G)\ne \emptyset$. We now prove the claim. First, note that $v_H\in f_{\text {pr}}(G')$ for the same reason as before, since $\delta ^-(v_H,G')=\delta ^-(v_H,G)$ and $\delta ^-(v,G')\le \delta ^-(v,G)$ for every $v\in V{\setminus } \{v_H\}$. Moreover, for every $v\in S^0{\setminus } \{v_H,v_L\}$ we have

$$\begin{aligned} \delta ^-(v,G')=\delta ^-(v,G)-1 = \delta ^-(v_H,G')-1= \Delta (G')-1 \end{aligned}$$

and $v<v_H$, so condition (b) in Lemma 1 does not hold for *v* and thus $v\notin f_{\text {pr}}(G')$. Analogously, for every $v\in S^1{\setminus } \{v_L\}$ we have

$$\begin{aligned} \delta ^-(v,G')=\delta ^-(v,G)-1 = \delta ^-(v_H,G')-2= \Delta (G')-2, \end{aligned}$$

so condition (b) in Lemma 1 does not hold for *v* and thus $v\notin f_{\text {pr}}(G')$. This allows to conclude the claim for $G'$. In the case of $G''$, we can write the following chain of inequalities,

$$\begin{aligned} (\delta ^-(\bar{v},G''),\bar{v})=(\delta ^-(\bar{v},G), \bar{v}) \succ (\delta ^-(v_H,G)-1, v_H) = (\delta ^-(v_H,G''), v_H), \end{aligned}$$

where the equalities hold because of the definition of $G''$ and the inequality by condition (b) in Lemma 1, given that $\bar{v}\in f_{\text {pr}}(G)$. Since $(v_H,\bar{v})\notin E$, we conclude from condition (a) in Lemma 1 that $v_H\notin f_{\text {pr}}(G'')$, and therefore the claim for $G''$ follows. This argument is illustrated in Fig. 4.

To see that the mechanism is impartial, we let $G=(V,E)\in \mathcal {G}_n,\ u\in f_{\text {prp}}(G)$, and $G'=(V,E')\in \mathcal {N}_u(G)$. We show in the following that $u\in f_{\text {prp}}(G')$, and since the graphs *G* and $G'$ are chosen arbitrarily, their roles can be inverted and this is enough to conclude that the mechanism is impartial. We first note that $u\in f_{\text {pr}}(G)$ because $f_{\text {prp}}(G)\subseteq f_{\text {pr}}(G)$, thus impartiality of asymmetric plurality with runners-up proven in Theorem 1 implies that $u\in f_{\text {pr}}(G')$. If $f_{\text {pr}}(G')=\{u\}$, then the condition in the mechanism holds trivially for this vertex, so that $u\in f_{\text {prp}}(G')$ and we conclude. Otherwise, let $v\in f_{\text {pr}}(G'){\setminus }\{u\}$ be an arbitrary vertex selected by asymmetric plurality with runners-up other than *u*. Since $u\in f_{\text {prp}}(G)$, we have that either (a) $v\notin f_{\text {pr}}(G)$, or (b) $v\in f_{\text {pr}}(G)$ and there exists $G_{uv} = (V, E_{uv}) \in \mathcal {N}_u(G)$ such that $v\notin f_{\text {pr}}(G_{uv})$. If (a) holds, taking $G'_{uv}=G$, which belongs to $\mathcal {N}_u(G')$ because of the assumption that $G' \in \mathcal {N}_u(G)$, we have that $v\notin f_{\text {pr}}(G'_{uv})$. If (b) holds, taking $G'_{uv}=G_{uv}$, which belongs to $\mathcal {N}_u(G')$ since $\mathcal {N}_u(G')=\mathcal {N}_u(G)$, we have that $v\notin f_{\text {pr}}(G'_{uv})$. In either case, we conclude that there exists $G'_{uv}\in \mathcal {N}_u(G')$ such that $v\notin f_{\text {pr}}(G'_{uv})$. Since this argument is valid for every $v\in f_{\text {pr}}(G'){\setminus }\{u\}$, we conclude that $u\in f_{\text {prp}}(G')$.

That asymmetric plurality with runners-up and pivotal vertices is 1-optimal is straightforward, since for every $G\in \mathcal {G}$ it selects a non-empty subset of $f_{\text {pr}}(G)$, and from Theorem 1 we know that this set contains vertices with indegrees in $\{\Delta (G), \Delta (G)-1\}$.

We finally show that the mechanism is decisive. Let $G=(V,E)\in \mathcal {G}_n$. This is immediate if $|f_{\text {pr}}(G)|\le n-1$ due to $f_{\text {prp}}(G)\subseteq f_{\text {pr}}(G)$. We thus suppose in what follows that $|f_{\text {pr}}(G)|=n$. In particular, Lemma 1 implies $(v,v_H)\in E$ for every $v\in V{\setminus } \{v_H\}$, thus $\Delta (G)=n-1$ and $\delta ^-(v,G)\ge n-2$ for every $v\in V$.

If $S^1=\emptyset$, then $\delta ^-(v)=n-1$ for every $v\in V$, i.e., *G* is the complete graph. In this case, $v_H=n$ and we claim that $v\notin f_{\text {prp}}(G)$ for each $v\in V{\setminus } \{n\}$, thus $|f_{\text {prp}}(G)|\le 1$. This comes from the fact that, for every $v\in V{\setminus }\{n\}$ and every $G'=(V,E')\in \mathcal {N}_v(G)$ it holds $n\in f_{\text {pr}}(G')$. To see this, fix $v\in V{\setminus }\{n\}$ and $G'=(V,E')\in \mathcal {N}_v(G)$ arbitrarily and note that $(n,u)\in E'$ for every $u\in V{\setminus } \{n\},\ \delta ^-(n,G')\ge n-2=\Delta (G')-1$, and $n>u$ for every $u\in V{\setminus } \{n\}$, so that Lemma 1 implies $n\in f_{\text {pr}}(G')$.

We now consider the case $S_1\ne \emptyset$ and suppose towards a contradiction that $f_{\text {prp}}(G)=V$. In this case, there is at least one vertex with outdegree at most $n-2$. Let *u* be an arbitrary vertex with $\delta ^+(u,G)\le n-2$, and let $\bar{v}\in S_1 {\setminus } \{u\}$ be the vertex with the highest index such that $(u,\bar{v})\notin E$, i.e., $\bar{v}=\max \{V{\setminus } (N^+(u,G) \cup \{u\} )\}$. Since $u\in f_{\text {prp}}(G)$, there exists a graph $G'=(V,E')\in \mathcal {N}_u(G)$ such that $\bar{v}\notin f_{\text {pr}}(G')$. From Lemma 1, this implies that there exists $\bar{w}\in V$ such that either (a) $(\delta ^-(\bar{w},G'),\bar{w})\succ (\delta ^-(\bar{v},G'),\bar{v})$ and $(\bar{v},\bar{w})\notin E'$, or (b) $\delta ^-(\bar{w},G')>\delta ^-(\bar{v},G')$ and $\bar{w}>\bar{v}$. Since $\bar{v}\in f_{\text {pr}}(G)$, we know from this same lemma that if (a) holds, $(\delta ^-(\bar{w},G),\bar{w})\prec (\delta ^-(\bar{v},G),\bar{v})$ because of having $\bar{w}\notin N^+(\bar{v},G)=N^+(\bar{v},G')$; and similarly, if (b) holds, $\delta ^-(\bar{w},G)\le \delta ^-(\bar{v},G)$ because of having $\bar{w}>\bar{v}$. In either case, since we have $\delta ^-(\bar{v},G)\le \delta ^-(\bar{v},G')$, we conclude that $\delta ^-(\bar{w},G')> \delta ^-(\bar{w},G)$ and, therefore $(u,\bar{w})\notin E$. If (a) holds, this is a contradiction because we would have $\{u,\bar{v}\}\cap N^-(\bar{w},G)=\emptyset$ and thus $\delta ^-(\bar{w},G)\le n-3$. If (b) holds, we reach a contradiction as well, because we would have $\bar{w}\in V{\setminus } N^+(u,G)$ and $\bar{w}>\bar{v}$, but we chose $\bar{v}$ to be the maximum of this set. $\square$

We have seen so far that impartiality and optimality are incompatible goals, but that there is an impartial mechanism that satisfies a slightly weaker version of optimality, namely selecting vertices with indegree either $\Delta$ or $\Delta -1$, while keeping the number of selected vertices under control. For some input graphs, both mechanisms we have introduced select only one vertex with maximum indegree and many vertices with indegree $\Delta -1$, and a natural question is whether one can avoid this, i.e., whether one can ensure to keep the number of selected vertices that are not maximum-indegree vertices under control as well. This may be a reasonable objective for applications where the credibility of a selection is threatened if not a majority of the selected vertices receive a maximal number of nominations. We finish this section by providing a negative answer to this question. The following theorem states that, whenever we restrict to graphs with maximum outdegree *d*, there are graphs for which any impartial and 1-optimal mechanism either does not select maximum-indegree vertices or selects at most one maximum-indegree vertex and at least $\big \lfloor \frac{d+1}{2} \big \rfloor$ vertices with indegree $\Delta -1$.

### Theorem 3

Let $n\in \mathbb {N}$, $n\ge 3$, and $d\in [n-1]$. Let *f* be a selection mechanism that is impartial and 1-optimal on $\mathcal {G}_n(d)$. Then, there exists $G=(V,E)\in \mathcal {G}_n(d)$ such that we either have $|f(G)\cap \{v\in V: \delta ^-(v,G)=\Delta (G)\}|=0$, or both

$$\begin{aligned} |f(G)\cap \{v\in V: \delta ^-(v,G)=\Delta (G)\}|&=1 \text { and}\\ |f(G)\cap \{v\in V: \delta ^-(v,G)=\Delta (G)-1\}|&\ge \bigg \lfloor \frac{d+1}{2} \bigg \rfloor . \end{aligned}$$

### Proof

Let *n* and *d* be as in the statement of the theorem. In the following, we suppose towards a contradiction that there is an impartial and 1-optimal selection mechanism *f* such that, for every $G\in \mathcal {G}_n(d)$, one of the following holds:

(i) $$|f(G)\cap \{v\in V: \delta ^-(v,G)=\Delta (G)\}|\ge 2$$, or
(ii) $$|f(G)\cap \{v\in V: \delta ^-(v,G)=\Delta (G)\}|=1$$,
  $$|f(G)\cap \{v\in V: \delta ^-(v,G)=\Delta (G)-1\}|< \big \lfloor \frac{d+1}{2} \big \rfloor $$.

**Fig. 5:**
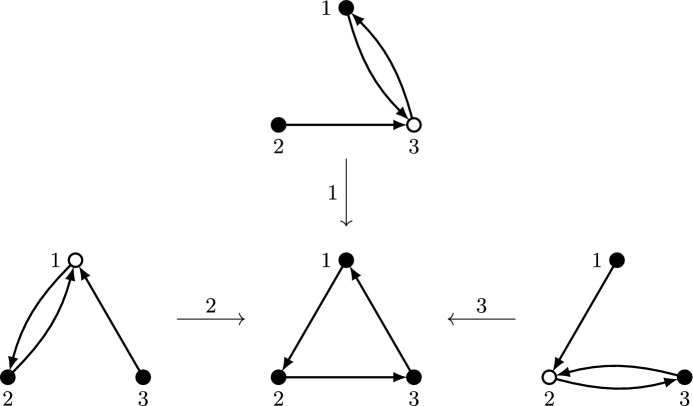
Counterexample to the existence of an impartial and 1-optimal selection mechanism that for every $G\in \mathcal {G}(d)$ satisfies (i) or (ii) as defined in the proof of Theorem 3, for $d\in \{1,2\}$. Vertices not shown in the figure have no incident edges. Vertices drawn in white have to be selected, vertices in black cannot be selected. For the graphs at the top, on the left, and on the right, this follows from (ii). An arrow with label *v* from one graph to another indicates that one can be obtained from the other by changing the outgoing edges of vertex *v*; by impartiality, the vertex thus has to be selected in both graphs or not selected in both graphs. It follows that no vertices are selected in the graph at the center, a contradiction to the claimed properties

We first prove the result for the case $d\in \{1,2\}$. We consider the graph $G=(V,E)\in \mathcal {G}_n(d)$ with $E=\{(1,2),(2,3),(3,1)\}$, consisting of a 3-cycle and $n-3$ isolated vertices. We consider as well, for $v\in \{1,2,3\}$, the graph $G_v=(V,E_v)\in \mathcal {G}_n(d)$ where *v* deviates from the 3-cycle by changing its outgoing edge to the previous vertex in the cycle, i.e.,

$$\begin{aligned}&E_1=\{(1,3),(2,3),(3,1)\},~E_2=\{(1,2),(2,1),(3,1)\},\\&\quad E_3=\{(1,2),(2,3),(3,2)\}; \end{aligned}$$

see Fig. 5 for an illustration. Since $|\{u\in V:\delta ^-(u,G_v)=\Delta (G_v)\}|=1$ for $v\in \{1,2,3\}$, (ii) must hold for each of these graphs, so $|f(G_v)\cap \{u\in V: \delta ^-(u,G_v)=\Delta (G_v)-1\}|=0$ for $v\in \{1,2,3\}$. In particular, this implies $v\not \in f(G_v)$ for $v\in \{1,2,3\}$. Since for each $v\in \{1,2,3\}$ it holds $E_v{\setminus } (\{v\}\times V)=E{\setminus } (\{v\}\times V)$, we conclude by impartiality that $v\notin f(G)$, and thus $f(G)\cap \{1,2,3\}=\emptyset$. This implies that neither (i) nor (ii) hold for graph *G*, a contradiction.

In the following, we assume $d\ge 3$. We denote $D=[d+1]$ and consider two families of graphs with *n* vertices, $K_v$ for each $v\in D$ and $K_{uv}$ for each $u,v\in D$ with $u\ne v$. They are constructed from a complete subgraph on *D* but deleting the outgoing edges of *v*, in the case of $K_v$, and the outgoing edges of *u* and *v*, in the case of $K_{uv}$. All the other vertices remain isolated. Formally, taking $V=[n]$ we define

$$\begin{aligned} K_v&= (V, (D{\setminus } \{v\})\times D) \text { for every } v\in D,\\ K_{uv}&= (V, (D{\setminus } \{u,v\})\times D) \text { for every } u,v\in D \text { with } u\ne v; \end{aligned}$$

**Fig. 6:**
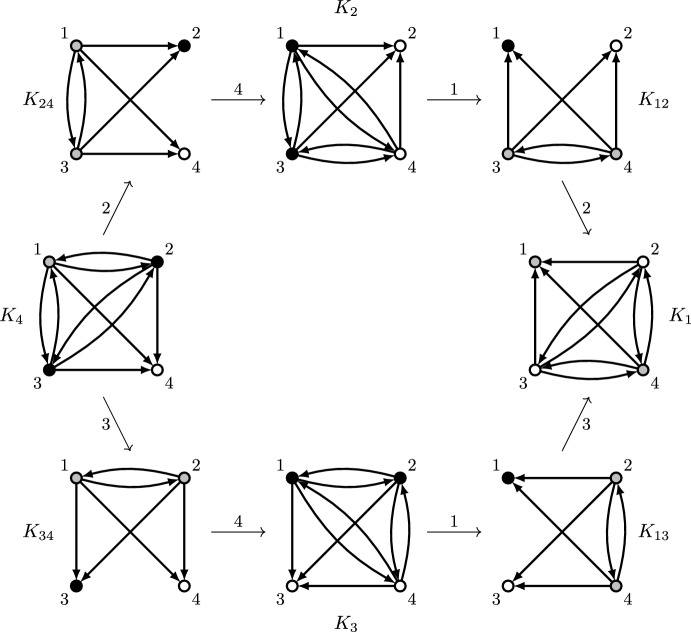
Counterexample to the existence of an impartial and 1-optimal selection mechanism that for every $G\in \mathcal {G}(3)$ satisfies (i) or (ii) as defined in the proof of Theorem 3. Vertices not shown in the figure have no incident edges. Vertices drawn in white have to be selected, vertices in black cannot be selected. For the graph on the left, this follows from (ii): under this assumption at most one of the vertices with indegree 2 can be selected, which without loss of generality we can assume to be vertex 1. For the other graphs, it then follows by impartiality and (i) or (ii). For example, in $K_{24}$ vertex 2 is not selected due to impartiality and vertex 4 is selected to satisfy (ii). Ultimately, we obtain a contradiction to the claimed properties for the graph on the right

see Fig. 6 for an illustration for $d=3$. Since $\{u\in V: \delta ^-(u,K_v)=\Delta (K_v)\} = \{v\}$ for every $v\in D$, (ii) must hold for every graph $K_v$ with $v\in D$. Specifically, for every $v\in D$ we have both $v\in f(K_v)$ and

2 $$\begin{aligned} |\{u\in D{\setminus } \{v\}: u\in f(K_v)\}| < \bigg \lfloor \frac{d+1}{2}\bigg \rfloor . \end{aligned}$$

Let $v\in D$ and $u\in D {\setminus } \{v\}$ be such that $u\notin f(K_v)$. Observing that

$$\begin{aligned} ((D{\setminus } \{v\})\times D){\setminus }(\{u\}\times V) = ((D{\setminus } \{u,v\})\times D){\setminus }(\{u\}\times V), \end{aligned}$$

we obtain from impartiality that $u\notin f(K_{uv})$. Since $\{w\in V: \delta ^-(w,K_{uv})=\Delta (K_{uv})\} = \{u,v\}$, this implies that (i) does not hold for $K_{uv}$, and (ii) yields $v\in f(K_{uv})$. Applying the fact that

$$\begin{aligned} ((D{\setminus } \{u,v\})\times D){\setminus }(\{v\}\times V) = ((D{\setminus } \{u\})\times D){\setminus }(\{v\}\times V), \end{aligned}$$

together with impartiality, we further obtain that $v\in f(K_u)$. We have shown the following property:

3 $$\begin{aligned} \text {For every } u,v\in D: \quad u\notin f(K_v) \Longrightarrow v\in f(K_u). \end{aligned}$$

Consider now the directed graph $H=(D,F)$, where for each $u,v\in D$ with $u\ne v$, we have $(u,v)\in F$ if and only if $u\notin f(K_v)$. Property (2) implies that

$$\begin{aligned} \delta ^-(v,H) > d-\bigg \lfloor \frac{d+1}{2}\bigg \rfloor \Longleftrightarrow \delta ^-(v,H) \ge d+1-\bigg \lfloor \frac{d+1}{2}\bigg \rfloor \end{aligned}$$

for each $v\in D$. Then, there has to be a vertex $v^*\in D$ such that $\delta ^+(v^*,H) \ge d+1-\big \lfloor \frac{d+1}{2} \big \rfloor$ holds as well. For this vertex, we have

$$\begin{aligned} \delta ^+(v^*,H) + \delta ^-(v^*,H) \ge 2\bigg (d+1-\bigg \lfloor \frac{d+1}{2}\bigg \rfloor \bigg ) \ge d+1. \end{aligned}$$

Since *H* has $d+1$ vertices, this implies the existence of $w^*\in D$ for which $\{(v^*,w^*),(w^*,v^*)\}\subset F$, i.e., both $v^*\notin f(K_{w^*})$ and $w^*\notin f(K_{v^*})$. This contradicts (3), so we conclude the proof of the theorem. $\square$

It is worth pointing out that the proof of the impossibility result when $d\ge 3$ uses graphs in which some vertices, in particular those with maximum indegree, do not have any outgoing edges. However, the impossibility extends naturally to the case where this cannot happen, corresponding to the practically relevant case in which abstentions are not allowed. For this, it is enough to define $D=[d]$ and modify each of the graphs $K_v$ and $K_{uv}$ considered in the previous proof in two ways: first, by adding a new vertex with outgoing edges to every vertex in *D* and incoming edges from the vertices in *D* which do not have any outgoing edge; second, by adding edges from each vertex in $V{\setminus } D$ to each vertex in *D*. The rest of the proof is completely analogous.

Our study in this section has focused on extensions of plurality with runners-up to a setting with outdegrees larger than one providing strong guarantees in terms of additive deviations from the maximum indegree, but losing symmetry properties of the original mechanism. The exploration of mechanisms treating agents symmetrically in this general setting, in line with the characterization of plurality with runners-up by Tamura (2016) for the outdegree-one case, constitutes a natural direction for future work.

## Trading off quantity and quality

We have so far given impartial and 1-optimal selection mechanisms for settings where the maximum outdegree *d* is smaller than the maximum number *k* of vertices that can be selected, and have shown that 1-optimality cannot be improved in such settings, in the sense that we cannot select only maximum-indegree vertices or bound the number of selected vertices which are not maximum-indegree vertices in general. We will now consider settings where $d\ge k$, such that asymmetric plurality with runners-up selects too many vertices and therefore cannot be used directly. In order to provide positive results for this setting, we need to relax 1-optimality and select vertices with lower, but still bounded, indegree. We obtain the following result.

### Theorem 4

For every $n \!\in \! \mathbb {N}$ and $k \!\in \! \{2,\ldots ,n\}$, there exists a selection mechanism that is impartial, *k*-decisive, and $\big (\big \lfloor \frac{n-2}{k-1}\big \rfloor +1\big )$-optimal on $\mathcal {G}_n$.

The result is obtained by a variant of asymmetric plurality with runners-up in which some edges are deleted before the mechanism is run. In principle, deleting a certain number of edges can affect the additive guarantee by the same amount, if all of the deleted edges happen to be directed at the same vertex. By exploiting the structure of the set of vertices selected by the mechanism, we will instead be able to delete edges to distinct vertices and thus keep the negative impact on the additive guarantee under control.

**Fig. 7:**
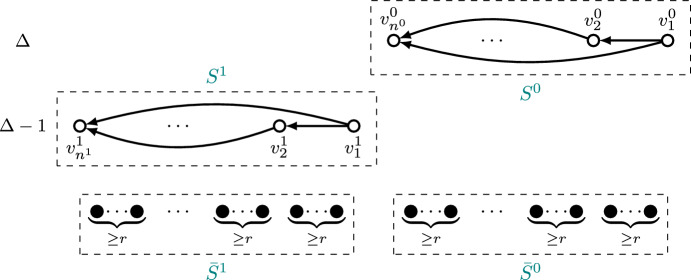
Illustration of Lemma 2. Vertices are arranged horizontally according to index, so that vertices on the left are favored in case of ties. Vertices in $S^0$ and $S^1$ are also arranged vertically according to indegree. Edges from vertices in $S^1$ to every vertex in $S^0$ have been omitted for clarity. There are no edges from a vertex to any of the *r* vertices to its left, which implies that for each vertex in $S^0$ or $S^1$, except for the left-most vertex, there are at least *r* vertices out of these sets

The modified mechanism, which we call *asymmetric plurality with runners-up and edge deletion*, is formally described in Algorithm 4. It deletes any edges from a vertex to the $\big \lfloor \frac{n-2}{k-1}\big \rfloor$ vertices preceding that vertex in the tie-breaking order, and applies asymmetric plurality with runners-up to the resulting graph. The following lemma, illustrated in Fig. 7, shows that without such edges, the maximum number of vertices selected is reduced to *k*.

**Algorithm 4:**
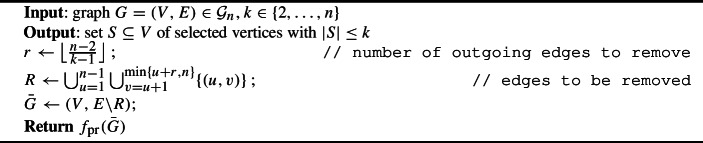
Asymmetric plurality with runners-up and edge deletion ($f_{\text {prd}}$)

### Lemma 2

Let $n\in \mathbb {N},\ k\in \{2,\ldots ,n\}$, and $r\in \mathbb {N}$ with $r\ge \big \lfloor \frac{n-2}{k-1}\big \rfloor$. Let $G=(V,E)\in \mathcal {G}_n$ be such that for every $u\in [n-1]$ and every $v\in \{u+1,\ldots , \min \{u+r,n\}\},\ (u,v)\notin E$. Then, $|f_{\text {pr}}(G)|\le k$.

### Proof

As in the proof of Theorem 2, we define $S^i=\{v\in f_{\text {pr}}(G):\delta ^-(v)=\Delta (G)-i\}$ and $n^i=|S^i|$ for $i\in \{0,1\}$, and now we denote its elements in increasing order by $v^i_j$ for $j\in [n^i]$, i.e.,

$$\begin{aligned} S^i=\{v^i_j\}_{j=1}^{n^i} \text { with } v^i_1<v^i_2\dots <v^i_{n^i} \text { for each } i\in \{0,1\}. \end{aligned}$$

From Lemma 1, we know that $f_{\text {pr}}(G)= S^0\cup S^1$, that for $i\in \{0,1\}$ we have $(v^i_j,v^i_k)\in E$ for every *j*, *k* with $j<k$, and that $v^1_{1}>v^0_{n^0}$. This allows to define, for $i\in \{0,1\}$,

$$\begin{aligned} {\bar{S}^i=\{v\in V{\setminus } S^i: v^i_1<v<v^i_{n^i}\},\quad \bar{n}^i=|\bar{S}^i|,} \end{aligned}$$

such that $$\bar{S}^0\cap \bar{S}^1=\emptyset $$.

Fix $i\in \{0,1\}$ and suppose that $n^i\ge 2$. Combining the facts that $(v^i_j,v^i_k)\in E$ for every *j*, *k* with $j<k$, and that $(u,v)\notin E$ for every $u\in [n-1]$ and $v\in \{u+1,\ldots ,\min \{u+r,n\}\}$, we have that for every $j\in [n^i-1]$ it holds $v^i_{j+1}-v^i_{j}\ge r+1$. Summing over *j* yields $v^i_{n^i}-v^i_1\ge (n^i-1)(r+1)$, hence

$$\begin{aligned} \bar{n}^i=v^i_{n^i}-v^i_1+1-n^i \ge (n^i-1)(r+1)+1-n^i =(n^i-1)r, \end{aligned}$$

where the first equality comes from the definition of the set $\bar{S}^i$. This yields $n^i\le 1+ \frac{\bar{n}^i}{r}$. We can now lift the assumption $n^i\ge 2$, since when $n^i\in \{0,1\}$ we have $\bar{n}^i=0$ and the inequality holds as well. We obtain the following chain of inequalities:

$$\begin{aligned} |f_{\text {pr}}(G)|=n^0+n^1 \le 2+\frac{\bar{n}^0+\bar{n}^1}{r} \le 2+\frac{n-|f_{\text {pr}}(G)|}{r}, \end{aligned}$$

where the last inequality comes from the fact that all the sets $S^0,\ S^1,\ \bar{S}^0,\ \bar{S}^1$ are disjoint and therefore their cardinalities sum up to at most *n*. This bounds the number of selected vertices as $|f_{\text {pr}}(G)|\le \frac{2r+n}{r+1}$.

Suppose now that $|f_{\text {pr}}(G)|\ge k+1$. Using the previous bound, this yields

$$\begin{aligned} 2r+n \ge (k+1)(r+1) \Longleftrightarrow r \le \frac{n-k-1}{k-1} = \frac{n-2}{k-1}-1, \end{aligned}$$

which contradicts the lower bound on *r* in the statement of the lemma. $\square$

We are now ready to prove Theorem 4.

### Proof of Theorem 4

We show that asymmetric plurality with runners-up and edge deletion satisfies the conditions of the theorem. Let $n\in \mathbb {N}$ and $k\in \{2,\ldots ,n\}$. Impartiality follows from the fact that asymmetric plurality with runners-up is impartial, thus the potential deletion of outgoing edges of a given vertex cannot affect the fact of selecting this vertex or not. Formally, if $G=(V,E),\ v\in V$ and $G'=(V,E')\in \mathcal {N}_v(G)$, then defining $\bar{G}=(V,\bar{E})$ and $\bar{G}'=(V,\bar{E}')$ as the graphs constructed when running asymmetric plurality with runners-up and edge deletion with *G* and $G'$ as input graphs, respectively, we have

$$\begin{aligned} \bar{E}{\setminus } (\{v\}\times V)&= (E{\setminus } (\{v\}\times V)){\setminus } \Bigg (\bigcup _{u=1}^{n-1}\bigcup _{w=u+1}^{\min \{u+r,n\}}\{(u,w)\}\Bigg )\\&= (E'{\setminus } (\{v\}\times V)){\setminus } \Bigg (\bigcup _{u=1}^{n-1}\bigcup _{w=u+1}^{\min \{u+r,n\}}\{(u,w)\}\Bigg )\\&= \bar{E}'{\setminus } (\{v\}\times V), \end{aligned}$$

where we use that $G'\in \mathcal {N}_v(G)$. Impartiality then follows directly from the impartiality of asymmetric plurality with runners-up. For the following, fix $G=(V,E)\in \mathcal {G}_n$ and define *r* and $\bar{G}=(V,E{\setminus } R)$ as in the mechanism. That asymmetric plurality with runners-up and edge deletion is a selection mechanism follows directly from the fact that asymmetric plurality with runners-up is a mechanism, due to $f_{\text {prd}}(G)=f_{\text {pr}}(\bar{G})$. Since the first step of the mechanism ensures that for every $u\in [n-1]$ and every $v\in \{u+1,\ldots , \min \{u+r,n\}\}$ we have $(u,v)\notin E{\setminus } R$, Lemma 2 implies that $|f_{\text {prd}}(G)|=|f_{\text {pr}}(\bar{G})|\le k$, i.e., the mechanism is *k*-decisive. Finally, in order to show $\alpha$-optimality for $\alpha =\big \lfloor \frac{n-2}{k-1}\big \rfloor +1$ we first note that, for every $v\in V,\ \delta ^-(v, G) \le \delta ^-(v, \bar{G})+r$, since at most $|\{v-r,\ldots ,v-1\}\cap V|\le r$ incoming edges of *v* are deleted when defining $\bar{G}$ from *G*. In particular, $\Delta (G) \le \Delta (\bar{G})+r$. Using this observation and denoting by $v^*\in \arg \min \{\delta ^-(v,G): v\in f_{\text {prd}}(G)\}$ an arbitrary element with minimum indegree among those selected by asymmetric plurality with runners-up and edge deletion, we obtain that

$$\begin{aligned} \delta ^-(v^*,G) \ge \delta ^-(v^*,\bar{G}) \ge \Delta (\bar{G})-1 \ge \Delta (G)-r - 1, \end{aligned}$$

where the second inequality comes from Lemma 1, since $v^*$ belongs to $f_{\text {pr}}(\bar{G})$. We conclude that the mechanism is $(r+1)$-optimal for $r=\big \lfloor \frac{n-2}{k-1}\big \rfloor$. $\square$

It is easy to see that the previous analysis is tight in terms of $\alpha$-optimality from a graph $G=(V,E)$ where exactly $r=\big \lfloor \frac{n-2}{k-1}\big \rfloor$ incoming edges of the maximum-indegree vertex are deleted, and a vertex with the second highest indegree *u* such that $u>\text {top}(G),\ (u,\text {top}(G))\in E$, and $\delta ^-(u)=\Delta (G)-r-1$ is selected. However, we do not know whether the tradeoff between the values of $\alpha$ and *k* for which an impartial mechanism can be $\alpha$-optimal and *k*-decisive provided by Theorem 4 is best possible, and the question for the optimum tradeoff is an interesting one. Currently, when $d\ge k$ a gap on the best possible value of $\alpha$ for which an impartial and *k*-decisive selection mechanism can be $\alpha$-optimal remains between the upper bound of $\big \lfloor \frac{n-2}{k-1}\big \rfloor +1$ and a lower bound of 1, which is relatively large when the number *k* of vertices that can be selected is small. We may, alternatively, also ask for the number of vertices that have to be selected in order to guarantee 1-optimality. Currently, the best upper bound on this number is $n-1$.

## Conclusion

We studied impartial mechanisms selecting multiple agents in a setting where each agent may cast up to a given number of *d* nominations for other agents. Our goal was to quantify the performance of such mechanisms by two numbers: We call a mechanism $\alpha$-optimal if each selected agent is lacking at most $\alpha$ nominations compared to the most nominated agent, and we call it *k*-decisive if it selects at most *k* agents. We have shown that there is no 0-optimal impartial mechanism, but that there exist 1-optimal and *k*-decisive impartial mechanisms whenever $k \ge \min \{d+1,n-1\}$. We further provided a mechanism that is impartial, *k*-decisive and $(\lfloor \frac{n-1}{k-1}\rfloor +1)$-optimal for any number *d* of nominations that each agent may cast. Finding mechanisms with a better trade-off between optimality and decisiveness is an interesting open for future research.

There may be more fine-grained measures for the performance of mechanisms beyond $\alpha$-optimality. For instance, among all 1-optimal mechanisms, one may be interested in finding a mechanism that selects many agents with the maximal number of nominations compared to the number of agents with one less nomination. However, we showed that every 1-optimal impartial mechanism will either sometimes select no agent with a maximal number of nominations, or will sometimes select only one such agent while selecting also more than *d*/2 agents with one less nomination.

Finally, we note that the mechanisms that we proposed in this paper sometimes do not select vertices with indegree strictly higher than the one of other selected vertices which may seem unfair. Unfortunately, this is unavoidable for any decisive mechanism, as the following simple proposition states.

### Proposition 2

Let $n\in \mathbb {N}$ and let *f* be a selection mechanism that is both impartial and decisive on $\mathcal {G}_n$. Then, there exists a graph $G=(V,E)\in \mathcal {G}_n$ and vertices $u,v\in V$ such that $\delta ^-(u)>\delta ^-(v)$ and $f(G) \cap \{u,v\} = \{v\}$.

### Proof

Fix *n* and *f* as in the statement and consider the complete graph $G=(V,E)$, where *E* is the set of all pairs of distinct vertices in *V*. Since *f* is decisive on $\mathcal {G}_n$, there exists $u\in V{\setminus } f(G)$. Let now $G'=(V,E')$ be the graph obtained by removing all outgoing edges of *u*; i.e., $E'=E{\setminus } (\{u\} \times V)$. Impartiality and the fact that $G'\in \mathcal {N}_u(G)$ imply $u\notin f(G')$. Since *u* has indegree $n-1$ and all other vertices have indegree $n-2$, the result follows for graph $G'$ by taking any vertex $v\in f(G')$. $\square$

## Footnotes

1 It is worth emphasizing that impartiality only prevents an agent from affecting its own selection, but not the number of agents that are selected. Tamura and Ohseto (2014) showed that a stricter notion of impartiality that also forbids the latter is incompatible with the basic axioms of positive and negative unanimity, as in the single-selection setting.

2 Our results still hold in the more general setting where we allow not to select any vertex sometimes and we define the selected indegree in these cases to be 0. We stick to mechanisms that always select for simplicity.

3 In this mechanism and wherever ties are broken in the rest of the paper, we break ties in favor of larger index, so $\text {top}(G)$ is the vertex with maximum indegree and highest priority in graph *G*. Naturally, any other deterministic tie-breaking rule could be used, as long as it only depends on the labels of the vertices and not on the nominations.
